# Idiopathic intracranial hypertension presenting as bilateral spontaneous lateral intrasphenoidal and transethmoidal meningoceles: a case report and review of the literature

**DOI:** 10.1186/s13256-018-1959-6

**Published:** 2019-03-05

**Authors:** Aleksandar Radonjic, Abdul Mounem Kassab, Ioana D. Moldovan, Shaun Kilty, Fahad Alkherayf

**Affiliations:** 1 Division of Neurosurgery, Department of Surgery, The Ottawa Hospital, Civic Campus, 1053 Carling Avenue, Room C2218, Ottawa, Ontario K1Y 4E9 Canada; 2 Department of Otolaryngology – Head & Neck Surgery, The Ottawa Hospital, Ottawa, Canada; 3 Faculty of Medicine, University of Ottawa, Ottawa, Canada; 4 The Ottawa Hospital Research Institute, Ottawa, Canada

**Keywords:** Bilateral, Spontaneous, Meningocele, Lateral intrasphenoidal, Transethmoidal, Expanded endoscopic, Endonasal, Surgery, Skull base, Case report

## Abstract

**Background:**

Basal meningoceles are rare herniations of the meninges that tend to present unilaterally with cerebrospinal fluid rhinorrhea. Growing evidence suggests that intracranial hypertension contributes considerably to the formation of spontaneous basal meningoceles.

**Case presentation:**

A 50-year-old man of Middle East ethnicity presented with a 16-week history of cerebrospinal fluid rhinorrhea, short-term memory loss, and slight decline in cognitive function. We present a case of bilateral spontaneous meningoceles with bone defects in the left lateral sphenoid sinus and right anterior cribriform plate, as well as with a remodeled sella. A neuronavigation-assisted expanded endoscopic endonasal surgery was performed to resect the meningoceles. Postoperative imaging demonstrated complete resolution of the bilateral meningoceles.

**Conclusions:**

This case reports the first bilateral basal spontaneous meningoceles in the literature. Furthermore, based on this case’s imaging results and the literature reviewed, elevated intracranial pressure may be a determining factor behind the development of spontaneous meningoceles.

## Background

Basal meningocele is a herniation of the meninges through a defect in the bone of the skull base. This disorder almost invariably presents with cerebrospinal fluid (CSF) rhinorrhea, and the clinical history may also include headache, vertigo, seizures, and meningitis [1]. The etiology behind spontaneous forms of this disorder has been debated; however, recent evidence points to increased intracranial pressure (ICP) as a driving cause [2]. Most spontaneous basal meningoceles present unilaterally with CSF rhinorrhea in adults. We present a case of bilateral spontaneous left lateral intrasphenoidal and right transethmoidal meningoceles in a 50-year-old man. This is a rare finding in which two types of skull base lesions present concurrently in an adult patient with no previous history of nasal surgery or trauma.

## Case presentation

A 50-year-old man of Middle East ethnicity presented with a 16-week history of CSF rhinorrhea, short-term memory loss, and slight decline in cognitive function. On physical examination, clear watery rhinorrhea, right-beating nystagmus, tongue deviation to the left side, mild facial asymmetry, multiple lipomas, bradycardia (52 beats/minute), and high blood pressure (194/118 mmHg) were detected. Laboratory tests results revealed presence of beta-2 transferrin in rhinorrhea fluid and hypokalemia (3 mmol/L). There were no other abnormalities in his hematology (for example, blood count) and chemistry test results (for example, liver function and CSF analysis). His past medical history was significant for: hypertension; Dercum’s disease; right internal carotid dissection with pseudoaneurysm formation which was stable and conservatively treated, and followed with imaging; chronic compensated noncommunicating hydrocephalus secondary to obstruction at aqueduct of Sylvius, and a one-time seizure episode.

**Fig. 1:**
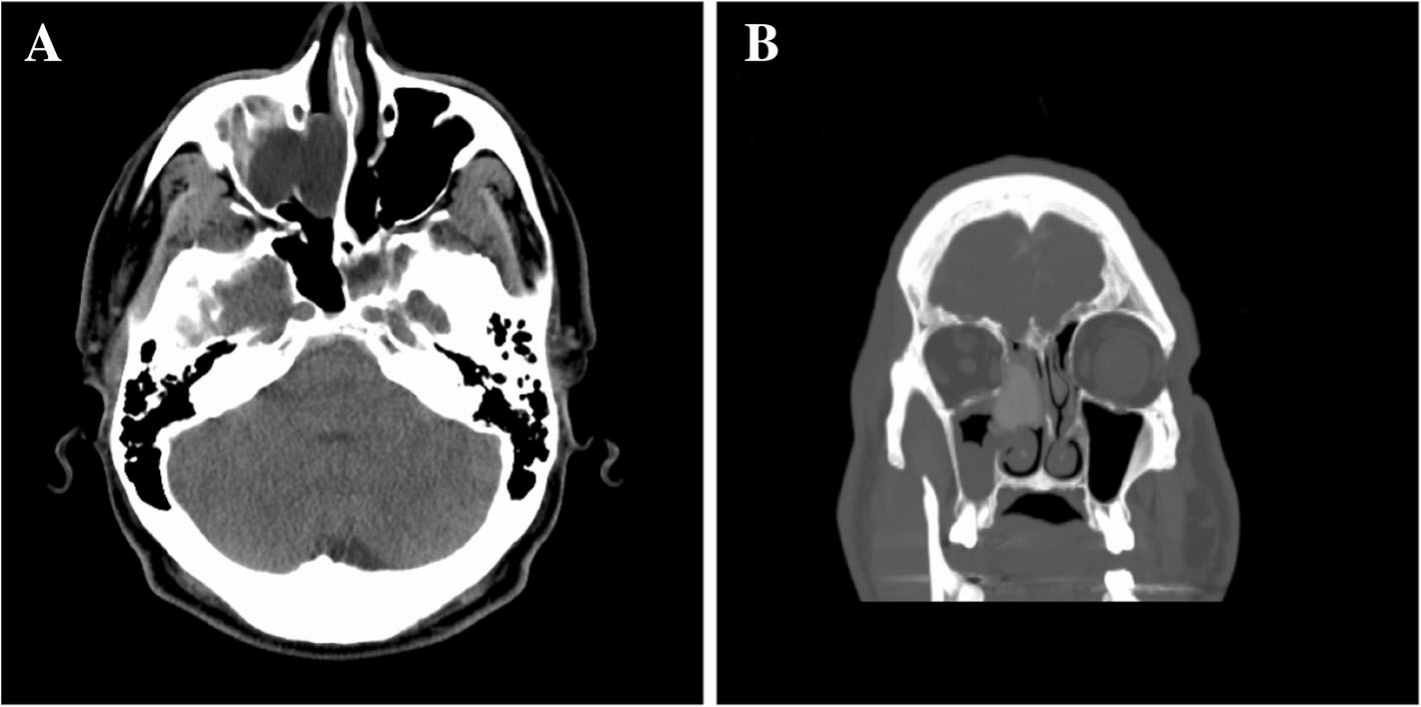
Computed tomography without contrast, preoperative images. **a** Axial and **b** coronal computed tomography images showed bony defects in the left lateral sphenoid sinus and right anterior cribriform plate

Computed tomography (CT) showed bony defects in his left lateral sphenoid sinus and right anterior cribriform plate (Fig. 1). CT cisternography revealed adjacent meningocele to the aforementioned defects with pooling of intrathecal contrast, confirming herniation into the left lateral sphenoid and right anterior ethmoid air cells.

**Fig. 2:**
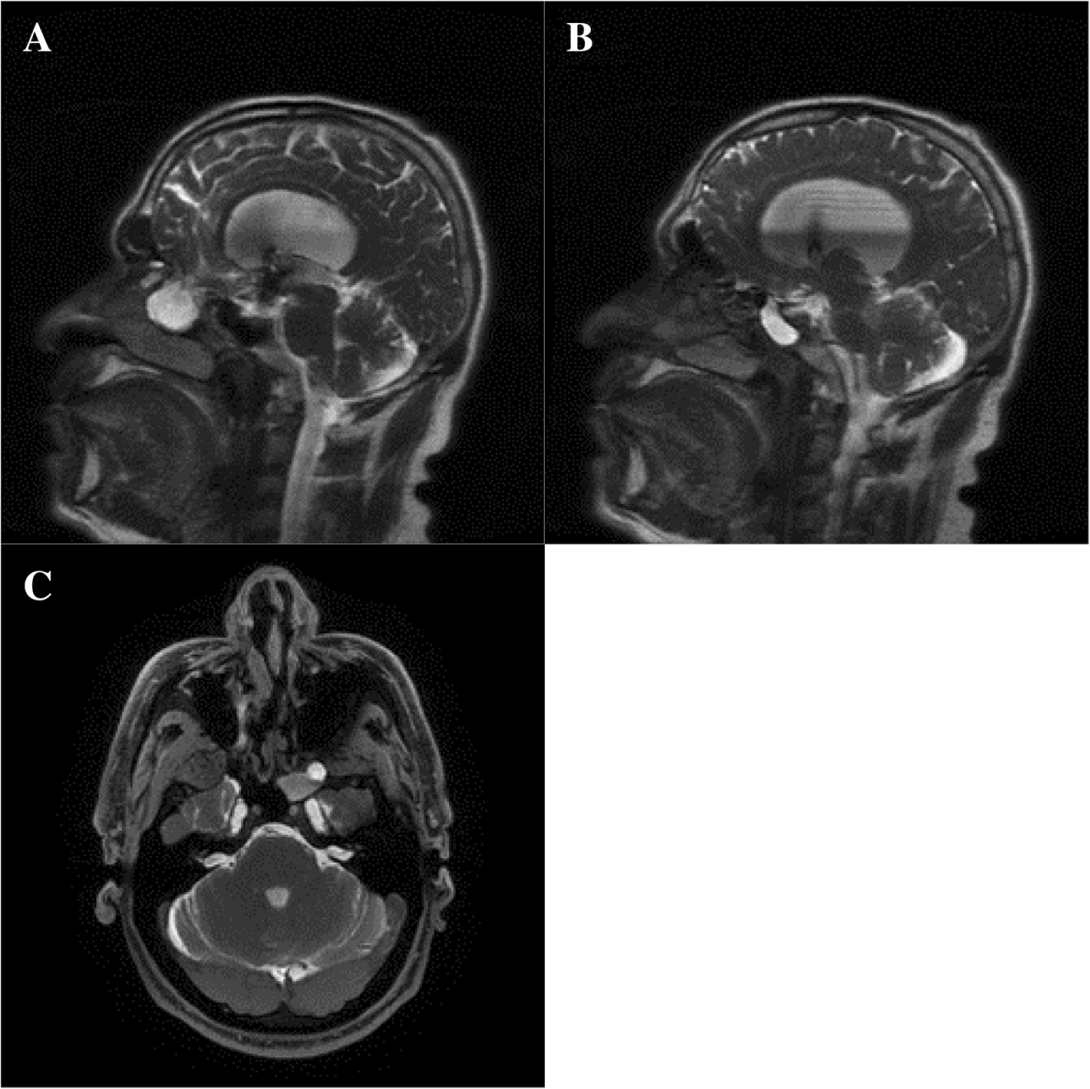
Magnetic resonance imaging cisternography, preoperative images. **a** and **b** Sagittal magnetic resonance imaging cisternography images showing right anterior ethmoidal (**a**) and left sphenoidal (**b**) meningoceles. **c** Axial magnetic resonance imaging cisternography image showing left sphenoidal meningocele

Magnetic resonance imaging (MRI) demonstrated a 2.9 × 1.8 × 1.8 cm right anterior meningocele traversing the anterior cribriform plate inferiorly into anterior ethmoid air cells and nasopharynx with extension into the right maxillary sinus (Fig. 2a). Another contrast extension from the left middle cranial fossa along its most anterior aspect into the most lateral aspect of the sphenoid sinus was identified suggesting a second meningocele measuring 1 × 1 × 0.9 cm (Fig. 2b). Both lesions were enhanced with gadolinium but no brain parenchyma could be identified within the sacs. Other findings on MRI included a significantly flattened pituitary gland within a remodeled sella and a slightly dilated ventricular system.

**Fig. 3:**
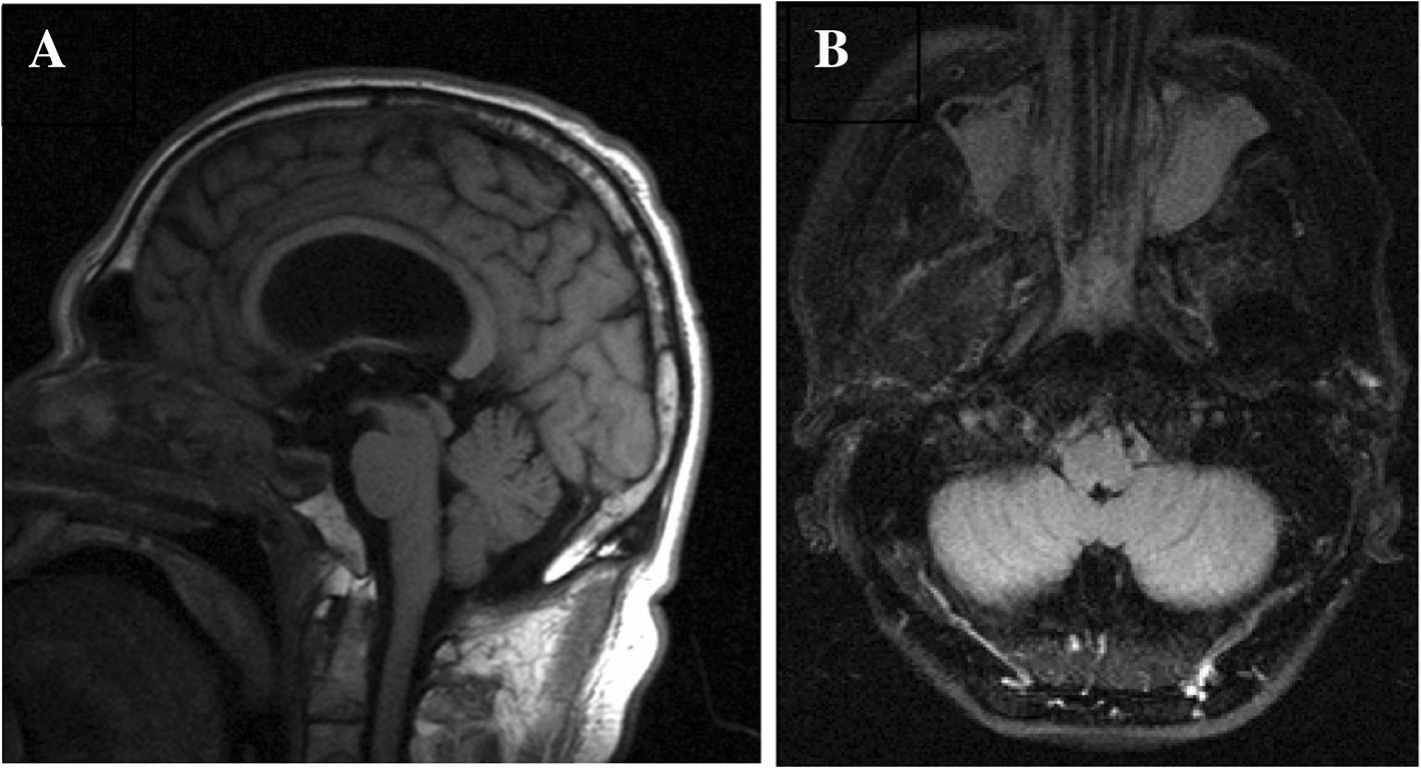
Magnetic resonance imaging without contrast, postoperative images. **a** Sagittal and **b** axial magnetic resonance imaging postoperative images showed no evidence of residual meningoceles

He underwent neuronavigation-assisted expanded endoscopic endonasal surgery with resection of the anterior skull base meningoceles. The first lesion was right ethmoidal and the second lesion was left sphenoidal. Repair of the dura was carried out with two layers of dural matrix. Insertion of a lumbar drain was done to drain CSF and for injection of fluorescein to help confirm dural seal. Opening pressure upon insertion of the lumbar drain at the time of surgery was 20 mmHg. Septal and anterior ethmoidal flaps were used to support the repair of the sphenoid and anterior ethmoidal lesions, respectively. He recovered uneventfully and postoperative imaging showed complete resolution of the meningoceles bilaterally (Fig. 3).

Four weeks after the surgery, he presented to our clinic with CSF leak and headache. MRI revealed evidence of CSF leak noted within the left sphenoid sinus. He underwent an endoscopic repair of the CSF leak and insertion of a ventriculoperitoneal shunt. Postoperation, he recovered well and presented no symptoms. He had 3-year follow up with no recurrence of the meningoceles.

## Discussion

We presented a rare case of a 50-year-old man with bilateral spontaneous lateral intrasphenoidal and transethmoidal meningoceles with nasal herniation and CSF rhinorrhea, associated with a significantly flattened pituitary gland within a remodeled sella.

Nasal meningoceles are herniations of the meninges into the nasal cavity [3]. Similar complications include encephaloceles (only the brain tissue is herniated) and meningoencephaloceles (both brain and meningeal tissues herniate). The location of the bone defect in the skull defines whether a meningocele is frontal, occipital, parietal, or basal [4].

The two types of basal meningoceles noted in this case report are transethmoidal and intrasphenoidal. Transsphenoidal lesions have been further classified as intrasphenoidal if the protrusion passes into but not through the sphenoid sinus [5]. There is some debate in the literature on whether intrasphenoidal defects are more common in the midline or the lateral recess [6]. Midline, perisellar sphenoid lesions were found to occur almost exclusively in obese women [6]. Pneumatization of the lateral sphenoid sinus, followed by pulsatile forces within the CSF, may lead to the formation of gaps in the bone found in lateral sphenoid lesions [7]. Our review of the literature found that 17 of 28 reported spontaneous intrasphenoidal lesions involved the lateral recess of the sphenoid sinus. Of those 17 lateral defects, 10 cases involved the right side (Table 1).

**Table 1:** Reported cases of spontaneous basal intrasphenoidal, transethmoidal, and bilateral herniations including meningoceles, encephaloceles, and meningoencephaloceles

Authors	Age, sex	Location	Type of herniation	Cerebrospinal fluid rhinorrhea?*	Bony defect
	*Transethmoidal*				
Sharifi *et al.* [10] 2014	35 F	Right	Meningoencephalocele	Yes	Ethmoid sinus
Hasegawa *et al.* [11] 2005	52 M	Right	Meningoencephalocele	Yes	Cribriform
Singh *et al.* [29] 2013	42 M	Left	Meningocele	No	Cribriform
Schwartz and Shaw [12] 2002	62 M	Left	Meningoencephalocele	Yes	Cribriform
Thijssen *et al.* [13] 1976	24 F	Left	Encephalocele	Yes	Cribriform
Ziade *et al.* [14] 2016	47.5 (median age) 8 Females 2 Males	3 left, 7 right	7 meningoceles, 3 meningoencephaloceles	Yes, all	Cribriform, all
	*Intrasphenoidal*				
Stefanelli *et al.* [15] 2014	41 M	Right	Meningoencephalocele	No	Greater wing of sphenoid
Kwon and Kim [16] 2010	45 F	Right	Meningoencephalocele	Yes	Pneumatized SS
Fraioli *et al.* [17] 2003	59 M	Right	Encephalocele	Yes	Lateral SS
Alfieri *et al.* [18] 2002	63 F	Right	Encephalocele	Yes	Lateral SS
Daniilidis *et al.* [19] 1999	46 F	Right	Encephalocele	Yes	Lateral SS
Deasy *et al.* [20] 1999	40 F, 59 M	Right	Encephalocele	Yes, both	Lateral SS
Clyde and Stechison [21] 1995	54 F	Right	Meningoencephalocele	Yes	Lateral SS
Peltonen *et al.* [22] 2008	60 M	Left	Encephalocele	Yes	Roof of SS
Herman *et al.* [23] 2003	45 F	Left	Meningoencephalocele	Yes	Ptosis, floor of third ventricle
Willner *et al.* [24] 1994	67 F	Left	Encephalocele	Yes	N/A
Albernaz *et al.* [25] 1991	47 F	Left	Encephalocele	No	Middle cranial fossa
Buchfelder *et al.* [26] 1987	44 F	Left	Encephalocele	Yes	Lateral SS
Sanjari *et al.* [8] 2013	45 F	Left	Meningocele	Yes	Lateral SS
Ogul *et al.* [27] 2014	42 F	N/A	Encephalocele	No	Hypomineralization of sphenoid bone
Abiko *et al.* [5] 1988	46 F	N/A	Encephalocele	No	Erosion of planum sphenoidale
Lai *et al.* [6] 2002	52.3 (mean age) 7 females, 5 males	7 left, 5 right	Encephaloceles (all)	3/12	8 lateral SS, 4 midline perisellar
	*Bilateral*				
Firat and Firat [28] 2004	53 M	Both	Meningoencephaloceles	Yes	Cribriform, lateral SS, anterior + posterior frontal sinus
Aggarwal *et al.* [2]2017	44 M	Both	Meningoencephaloceles	Yes	Bilateral DAVF
Schlosser and Bolger [9] 2002	49.2 (mean age) 4 females, 1 male	Both	Meningoencephaloceles (all 5)	4/5 yes	Bilateral lateral SS × 3, posterior ethmoid/frontal, frontal/central sphenoid

*DAVF* dural arteriovenous fistula, *F* female, *M* male, *N/A* not available, *SS* sphenoid sinus, * cerebrospinal fluid rhinorrhea at presentation

Spontaneous intrasphenoidal meningoceles, herniations limited to brain meninges, are rare; only one has been reported in the literature [8]. Most cases involve brain tissue and are generally unilateral [2, 5, 6, 9–28]. On the other hand, spontaneous transethmoidal lesions typically involve meningeal tissue, in the form of a meningocele or a meningoencephalocele [10–14, 29] (Table 1).

Our case reports a finding of bilateral meningoceles in an adult male involving both transethmoidal and lateral recess intrasphenoidal lesions. The bilateral nature of this lesion is a rare finding. As far as we know, no cases of bilateral, basal spontaneous meningoceles have been reported in the literature. However, seven cases of spontaneous bilateral meningoencephaloceles have been reported [2, 9, 28]. Of these, five patients had bilateral lateral recess intrasphenoidal meningoencephaloceles [2, 9, 28] (Table 1). No cases involved both transethmoidal and intrasphenoidal lesions. Thus, as far as we know, our case reports the first finding of bilateral basal spontaneous meningoceles in the literature. Furthermore, it depicts the first case of bilateral intrasphenoidal and transethmoidal defects with nasal herniation of any kind in the literature.

The etiology of basal meningoceles has historically been classed into congenital, iatrogenic, traumatic, and spontaneous causes [9]. Spontaneous cases almost invariably present as CSF rhinorrhea in adult patients [2, 6, 8–14, 16–24, 26, 28, 29]. The mean age of the 15 spontaneous transethmoidal reported cases was 46 at presentation [10–14, 29]. Only one of these patient’s histories did not include CSF rhinorrhea [29]. Similarly, the mean age of the 28 spontaneous intrasphenoidal reported cases was 51 with 15 showing signs of CSF rhinorrhea [5, 6, 8, 15–27] (Table 1). Traditionally, the spontaneous category was synonymous with idiopathic as it was reserved for CSF leaks that did not have a discernable cause [30]. However, recent literature strongly suggests that many spontaneous causes are the result of increased ICP [2]. One common sign of elevated ICP is empty sella syndrome. This is manifested radiologically as an empty sella due to compression of the pituitary gland as CSF replaces normal pituitary space [31]. Schlosser and Bolger found that all four of their patients with bilateral meningoencephaloceles had positive empty sella syndrome on radiography [9]. On MRI, we found a significantly flattened pituitary gland within a remodeled sella, indicative of empty sella syndrome as well. This finding, in the absence of congenital, tumor, or traumatic etiology, may strengthen the argument that elevated ICP is implicated in the formation of a spontaneous meningocele.

## Conclusion

Our case reports the first spontaneous bilateral left lateral intrasphenoidal and right transethmoidal meningoceles in an adult male. This case highlights evidence that elevated ICP may be a determining factor behind spontaneous meningoceles.
